# Pathological Vertebral Fracture Arising From Methotrexate-Associated Lymphoproliferative Disorder in a Patient With Systemic Lupus Erythematosus

**DOI:** 10.7759/cureus.40079

**Published:** 2023-06-07

**Authors:** Takaki Kitamura, Tomonori Shigemura, Yohei Yamamoto, Yasuaki Murata

**Affiliations:** 1 Orthopaedics, Chiba University, Graduate School of Medicine, Chiba, JPN; 2 Orthopaedics, Teikyo University Chiba Medical Center, Ichihara, JPN

**Keywords:** systemic lupus erythematosus, bone tumors, spinal fixation without fusion, mtx-lpd, pathological fracture

## Abstract

The incidence of lymphoproliferative disorders associated with methotrexate is rising in patients with rheumatoid arthritis. These disorders typically exhibit spontaneous tumor regression upon discontinuation of methotrexate therapy. Spinal lesions associated with these diseases are extremely rare. We present a case of systemic lupus erythematosus in which the patient developed lumbar spine lymphoproliferative disorders secondary to methotrexate therapy, which failed to regress despite discontinuation of the drug, ultimately leading to pathological fracture necessitating posterior spinal fixation. A 60-year-old woman had been diagnosed with systemic lupus erythematosus at the age of 55 years and had been taking prednisolone, hydroxychloroquine, and methotrexate. Throughout the course of her treatment, she experienced recurrent tumefaction and lymph node swelling in various locations. These masses and lymphadenopathy were believed to be potential complications of methotrexate-associated lymphoproliferative disorders, leading to the discontinuation of methotrexate. One month prior to cessation of methotrexate therapy, the patient presented to an orthopedic clinic with lower back pain, and T2-weighted magnetic resonance imaging revealed low signal intensity in the Th10 and L2 vertebrae, initially misdiagnosed as lumbar spinal stenosis. The patient was eventually referred to our department under suspicion of malignant pathology. Computed tomography identified a vertical fracture of the L2 vertebra, which, in conjunction with the imaging results, led to the diagnosis of pathological fracture secondary to methotrexate-associated lymphoproliferative disorder. Following admission to our department, bone biopsy and percutaneous pedicle screw fixation were performed one week later. Pathological examination confirmed the diagnosis of methotrexate-associated lymphoproliferative disorder. Given the possibility of pathological fracture in patients on methotrexate therapy experiencing severe back pain, additional imaging studies should be considered.

## Introduction

Methotrexate (MTX) is widely administered as a therapeutic intervention for rheumatoid arthritis and has demonstrated strong efficacy in the management of systemic lupus erythematosus (SLE) [1]. However, MTX use is associated with a variety of adverse effects, with recent studies indicating an increasing incidence of MTX-associated lymphoproliferative disorders (MTX-LPDs), particularly in patients with rheumatoid arthritis. [2]. These disorders often result in spontaneous tumor regression following the cessation of the drug [3]. Here, we present a case of SLE in a patient who developed MTX-LPD affecting the lumbar spine, which failed to regress after the discontinuation of MTX, ultimately resulting in a pathological fracture requiring posterior spinal fixation.

## Case presentation

The patient was a 60-year-old woman who had been diagnosed with SLE at the age of 55 years and had been taking prednisolone (PSL) at a dose of 5 mg/day and hydroxychloroquine at a dose of 200 mg/day since then. Quantitative evaluation of bone mineral density (BMD), facilitated by dual-energy X-ray absorptiometry (DXA), elucidated that her lumbar bone density approximated 74% of the respective young adult mean percentage (YAM%). The patient's hematological evaluation displayed a calcium level of 8.5 mg/dL and an alkaline phosphatase concentration of 110 U/L. Subsequently, she was administered a regimen of vitamin D supplementation. Due to the exacerbation of arthralgia, she was initiated on MTX at a dose of 6 mg/day at the age of 58 years, which was subsequently increased to 12 mg/day in response to inadequately managed articulatory discomfort. Two years subsequent to the implementation of MTX therapy, aberrant tissue growths manifested in the parotid and submandibular glands, in conjunction with inguinal lymphadenopathy. Simultaneously, an additional mass emerged proximate to the shoulder region. The histological analysis of a biopsy from the shoulder mass failed to yield evidence of malignant transformations and consequently resulted in a diagnostic interpretation of reactive vasculitis. Evaluation of hematological samples revealed the presence of viral capsid antigen (VCA)-IgM antibodies, as well as Epstein-Barr nuclear antigen (EBNA) antibodies, thereby unequivocally substantiating the existence of the Epstein-Barr virus (EBV). The obtained measurement of the soluble interleukin-2 receptor (sIL-2R) within the blood sample was found to be 895 U/ml. These masses at various sites and lymphadenopathy were considered potential complications of MTX-LPD. Consequently, MTX was prudently discontinued three years subsequent to the initiation of the therapeutic regimen. Subsequent positron emission tomography imaging demonstrated a diminution in the pervasiveness of lesions across multiple sites, a finding further corroborating the supposition of MTX-LPD in this patient.

**Figure 1:**
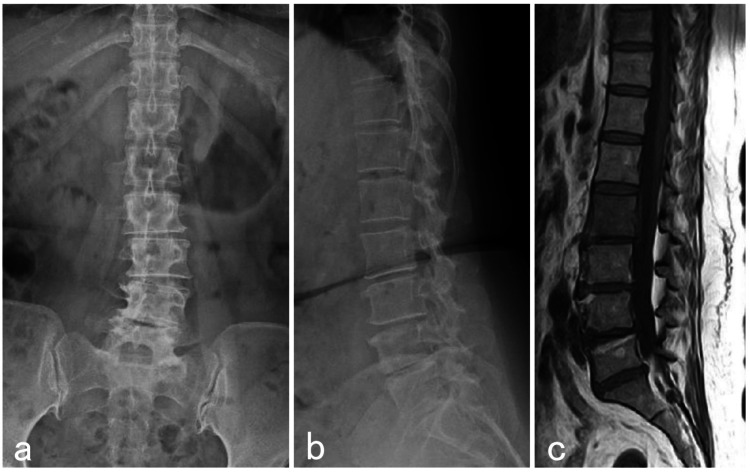
Initial imaging examination Anterior-posterior radiograph shows the lumbarization of S1 vertebrae (a). Lateral radiograph does not show vertebral fracture (b). The magnetic resonance imaging with a T1-weighted sagittal image reveals a hypointense change involving the T11 and L3 vertebral body (c).

One month prior to the cessation of MTX treatment, the patient initiated therapy at an orthopedic clinic for the onset of lower back pain with no history of traumatic injury. As the back pain persisted following the discontinuation of MTX, a plain radiograph and magnetic resonance imaging (MRI) were performed, and medication was initiated upon the diagnosis of lumbar spinal stenosis. The T1-weighted MRI image demonstrated low signal intensity in the Th11 and L3 vertebrae; however, the conservative treatment regimen was continued (Figures 1a-1c).

**Figure 2:**
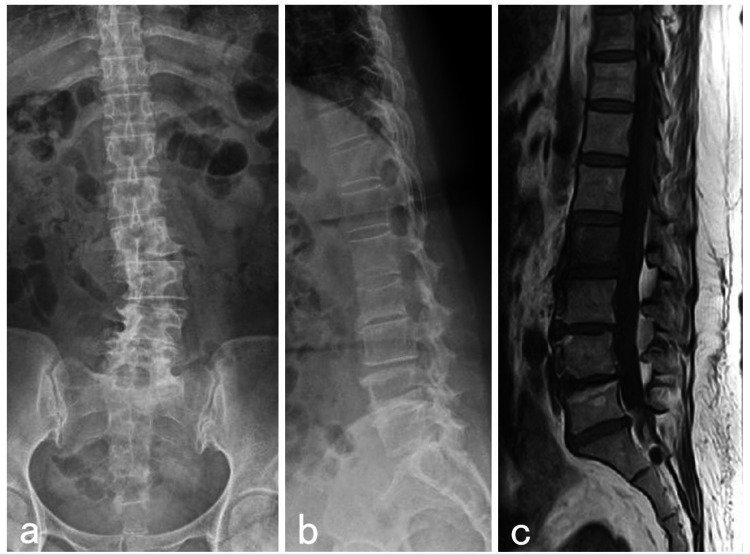
Second imaging examination Anterior-posterior radiograph shows a fracture of the inferior end plate of L3 vertebral body (a). Lateral radiograph shows wedge deformity of L3 vertebral body (b). The magnetic resonance imaging with a T1-weighted sagittal image reveals an expanded range of signal changes in the L3 vertebral body (c).

Two months before the presentation, the patient's back pain had worsened, and a subsequent MRI performed at another hospital revealed an expanded range of signal changes in the L3 vertebrae, leading to a referral to our department under the suspicion of malignant pathology (Figures 2a-2c).

**Figure 3:**
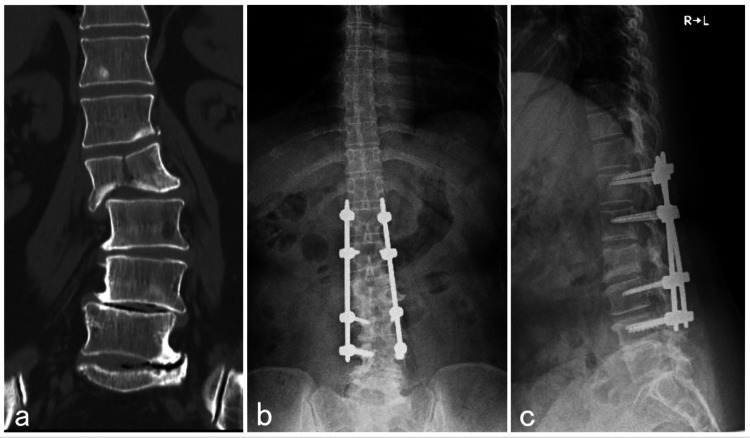
CT scan before surgery and plain radiograph after surgery The coronal CT scan shows a vertical fracture of the L3 vertebral body (a). Postoperative anteroposterior (b) and lateral (c) radiographs show pedicle screws were inserted from L1 to L5.

Physical examination revealed no muscle weakness, sensory disturbance, or abnormal tendon reflexes. However, the patient reported low back pain that was exacerbated by changes in posture. Computed tomography (CT) revealed a vertical fracture of the L3 vertebra, which, in conjunction with the MRI findings, led to the diagnosis of pathological fracture. Bone biopsy and percutaneous pedicle screw fixation were performed one week following admission to our department to identify the etiology and prevent paralysis resulting from fracture progression (Figures 3a-3b).

**Figure 4:**
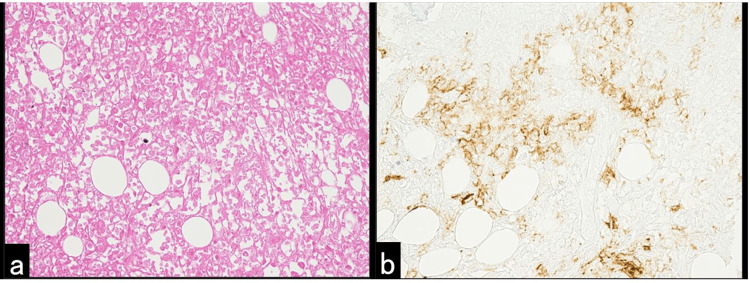
Pathological examination Histological findings on the L3 vertebra. The presence of ghost cells in the bone marrow indicates cancer. Hematoxylin and eosin staining (H & E) (a). Immunohistochemical analysis shows positive staining for CD20, indicating massive necrosis of B-cell lymphoproliferative disease. CD20 (b).

Rehabilitation was initiated immediately after surgery, and the patient was discharged from the hospital within two weeks. Pathological examination revealed the presence of ghost cells in the bone marrow, which were suspected to be indicative of massive necrosis of B-cell lymphoproliferative disease, characterized by the presence of CD45-positive cells, CD20-positive cells, and CD3-negative cells (Figures 4a, 4b).

**Figure 5:**
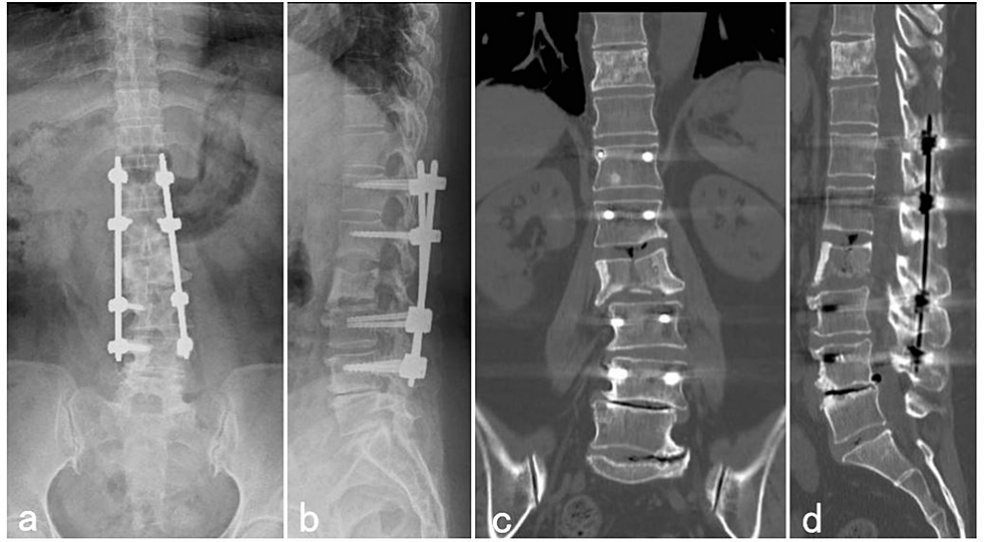
Plain radiograph and CT scan one year after surgery Postoperative anteroposterior (a) and lateral (b) radiographs show no loosening of the screws from L1 to L5 and osteosclerosis in T11 and L3 vertebral body. The coronal (c) and sagittal (d) CT imaging revealed complete osteosclerosis in the Th11 vertebral body and partial osteosclerosis and necrosis in the L3 vertebral body.

In conjunction with the clinical findings, this result of pathological examination led to a definitive diagnosis of MTX-LPD. However, the specific subtype of MTX-LPD could not be determined pathologically because of the extensive necrotic changes observed in the majority of specimens. Subsequent treatment for SLE included PSL and hydroxychloroquine. One year after surgery, the patient regained the ability to ambulate independently. Radiographic and CT imaging revealed complete osteosclerosis in the Th11 vertebral body, partial osteosclerosis, and necrosis in the L3 vertebral body, with the latter remaining in delayed union (Figures 5a-5d).

Despite these findings, no loosening of the screws occurred, and the patient reported a significant improvement in back pain. The patient has continued to be monitored on an outpatient basis for progressive compression of the vertebral body.

## Discussion

Herein, we describe the case of a patient with SLE who developed MTX-LPD in the lumbar spine. Despite the discontinuation of medication, the lesion failed to improve and a pathological fracture three months after cessation necessitated lumbar spine surgery. MTX-LPDs have been observed to occur in a variety of sites [4]; however, most reported cases have been in patients diagnosed with rheumatoid arthritis [2,3]. To the best of our knowledge, MTX-LPD has so far been reported in the elbow, hand, femur, and spine, and four cases of MTX-LPD in the spine have been documented [5-11]. MTX-LPD is characterized by spontaneous regression following the discontinuation of MTX, which occurs in approximately 60% of cases [12]. Patients prone to spontaneous regression tend to be characterized by EBV-positive cases and low soluble interleukin-2 receptor (sIL-2R) values [3]. In the present case, the patient was EBV positive and the sIL-2R value was relatively low. Although MTX-LPD was suspected at sites other than the spine, and these lesions regressed as a result of drug discontinuation, the MTX-LPD in the L3 vertebral body failed to regress, leading to a pathological fracture. If a patient taking MTX complains of back or leg pain and the pathology cannot be identified through radiography or other means, it may be necessary to suspect MTX-LPD in the spine at an early stage using MRI.

Pathological fractures of the lumbar spine resulting from osteonecrosis caused by MTX-LPD may also occur. Primary bone lymphoma can further lead to spinal fractures, and conservative management is typically the preferred approach in cases that do not result in neurological impairment. Percutaneous vertebroplasty or posterior spinal fusion may be used in cases with a risk of myelopathy. Chemotherapy or radiation therapy is frequently administered in conjunction with these treatments, either prior to or following the procedure [13]. Thus far, there have been no documented cases of pathological fractures of the spine caused by osteonecrosis following MTX-LPD. However, there has been one report of spontaneous necrosis of MTX-LPD in the liver, and a report of pathological fracture in the femur, necessitating open reduction and internal fixation, followed by chemotherapy has been documented [14,15]. In the latter report, X-rays performed one year postoperatively revealed an osteosclerotic image at the tumor site. However, the relationship between MTX-LPD and spontaneous necrosis remains unclear.

The patient in the present case was EBV positive and had other characteristics which might indicate spontaneous regression. However, owing to the presence of a pathological fracture, the patient underwent posterior fixation. As the other regions of the thoracic spine had previously regressed spontaneously, no additional chemotherapy was administered, with the expectation of spontaneous regression of the lumbar spine. Consequently, one year post-surgery, the site of the fracture in the L3 vertebral had yet to fully fuse but showed signs of partial osteosclerosis, and the patient reported an improvement in pain symptoms. The Th11 vertebral lesion also displayed complete osteosclerosis. Given that this is not a typical MTX-LPD as it does not exhibit complete regression, further monitoring is required.

## Conclusions

We encountered a patient with SLE who developed MTX-LPD of the lumbar spine, resulting in a pathological fracture despite the discontinuation of MTX treatment. Posterior spinal fixation was subsequently performed. In instances where a patient on MTX therapy presents with severe back pain, it is advisable to conduct additional imaging studies because of the possibility of pathological fracture in MTX-LPD. The mechanism underlying the persistence of MTX-LPD in the lumbar spine in this case and the subsequent development of a pathological fracture remains unclear. Further accumulation of cases of MTX-LPD in the spine is necessary to achieve a better understanding.
